# Estimating the fitness cost and benefit of cefixime resistance in *Neisseria gonorrhoeae* to inform prescription policy: A modelling study

**DOI:** 10.1371/journal.pmed.1002416

**Published:** 2017-10-31

**Authors:** Lilith K. Whittles, Peter J. White, Xavier Didelot

**Affiliations:** 1 Department of Infectious Disease Epidemiology, School of Public Health, Imperial College London, London, United Kingdom; 2 MRC Centre for Outbreak Analysis and Modelling, School of Public Health, Imperial College London, London, United Kingdom; 3 NIHR Health Protection Research Unit in Modelling Methodology, School of Public Health, Imperial College London, London, United Kingdom; 4 Modelling and Economics Unit, National Infection Service, Public Health England, London, United Kingdom; University of Bern, SWITZERLAND

## Abstract

**Background:**

Gonorrhoea is one of the most common bacterial sexually transmitted infections in England. Over 41,000 cases were recorded in 2015, more than half of which occurred in men who have sex with men (MSM). As the bacterium has developed resistance to each first-line antibiotic in turn, we need an improved understanding of fitness benefits and costs of antibiotic resistance to inform control policy and planning. Cefixime was recommended as a single-dose treatment for gonorrhoea from 2005 to 2010, during which time resistance increased, and subsequently declined.

**Methods and findings:**

We developed a stochastic compartmental model representing the natural history and transmission of cefixime-sensitive and cefixime-resistant strains of *Neisseria gonorrhoeae* in MSM in England, which was applied to data on diagnoses and prescriptions between 2008 and 2015. We estimated that asymptomatic carriers play a crucial role in overall transmission dynamics, with 37% (95% credible interval CrI 24%–52%) of infections remaining asymptomatic and untreated, accounting for 89% (95% CrI 82%–93%) of onward transmission. The fitness cost of cefixime resistance in the absence of cefixime usage was estimated to be such that the number of secondary infections caused by resistant strains is only about half as much as for the susceptible strains, which is insufficient to maintain persistence. However, we estimated that treatment of cefixime-resistant strains with cefixime was unsuccessful in 83% (95% CrI 53%–99%) of cases, representing a fitness benefit of resistance. This benefit was large enough to counterbalance the fitness cost when 31% (95% CrI 26%–36%) of cases were treated with cefixime, and when more than 55% (95% CrI 44%–66%) of cases were treated with cefixime, the resistant strain had a net fitness advantage over the susceptible strain. Limitations include sparse data leading to large intervals on key model parameters and necessary assumptions in the modelling of a complex epidemiological process.

**Conclusions:**

Our study provides, to our knowledge, the first estimates of the fitness cost and benefit associated with resistance of the gonococcus to a clinically relevant antibiotic. Our findings have important implications for antibiotic stewardship and public health policies and, in particular, suggest that a previously abandoned antibiotic could be used again to treat a minority of gonorrhoea cases without raising resistance levels.

## Abstract 2

In a modeling study, Xavier Didelot and colleagues address the implications of cefixime resistance for treatment of gonorrhea.

## Author summary

**Why was this study done?:**

- Gonorrhoea is an increasingly common sexually transmitted infection in England.
- Gonorrhoea has become resistant to many antibiotics in recent years, to the extent that clinicians are running out of treatment options.
- The current recommended treatment is a combination of 2 antibiotics, which is effective, but there are worrying signs that it may not always be so.
- Informed decisions need to be made to fight antibiotic-resistant gonorrhoea, but this is currently hampered by a lack of understanding of the dynamics of how resistance emerges and spreads.

**What did the researchers do and find?:**

- We developed a mathematical model that captures the dynamics of cefixime resistance observed between 2008 and 2015.
- The model includes a fitness benefit of resistance, to explain why cefixime resistance increased when cefixime was widely used up until 2010.
- The model also includes a fitness cost of resistance, to explain why cefixime resistance decreased from 2010.
- Having inferred the fitness cost and benefit of cefixime resistance, we were able to predict what would happen if cefixime was reintroduced and found that it could be used to treat up to 25% of gonorrhoea cases without risk.

**What do these findings mean?:**

- Cefixime had previously been abandoned because of a high level of resistance, but the fitness cost of resistance is such that resistance levels have since dropped, and reintroduction could be considered to treat a minority of cases.
- This policy would reduce the risk of resistance developing to the other antibiotics currently used.
- Similar modelling studies could be carried out to investigate the fitness costs and benefits of resistance to other antibiotics in gonorrhoea and other bacterial pathogens, providing a strong evidence basis for antibiotic deployment policies.
- In the future, there could be a policy of treating patients with gonorrhoea with a range of antibiotic regimens that would be systematically randomized at the individual patient level. The frequency of use of each regimen would be determined by the fitness costs and benefits of resistance to each regimen, once these have been measured.

## Introduction

Gonorrhoea, caused by the bacterial pathogen *N*. *gonorrhoeae*, is one of the most common sexually transmitted infections in England. Incidence has increased year on year since 2008, culminating in over 41,000 cases in 2015 [1]. Around 22,000 of these cases were found in men who have sex with men (MSM), constituting a 20% annual increase. The greatest cause for concern, however, is the rapid growth in antimicrobial resistance. The bacterium has quickly developed resistance to each first-line antibiotic in turn, from penicillin to cephalosporins, such as cefixime and ceftriaxone [2, 3]. Treatment with ceftriaxone is the last remaining single-drug option in most settings worldwide; however, diminishing susceptibility led England and many other countries to recommend treatment of gonorrhoea with a dual therapy of ceftriaxone and azithromycin [4–6]. Ceftriaxone resistance has been detected only sporadically in England; however, azithromycin resistance is easily selected for and was prevalent in a recent outbreak [7]. Resistance to azithromycin effectively reduces the current treatment to a monotherapy, making resistance trends increasingly important to monitor against the threat of potentially untreatable gonorrhoea.

Public Heath England (PHE) runs the Gonococcal Resistance to Antimicrobials Surveillance Programme (GRASP) [8], which has produced a report annually since 2000 [9–24]. GRASP monitors trends in resistance and susceptibility to a panel of antibiotics used to treat gonorrhoea in England and Wales and thus informs national treatment guidelines and strategy. In 2004, GRASP began testing for cefixime resistance, defined as having a minimum inhibitory concentration (MIC) of ≥0.125 mg/l [13]. A retrospective study of 133 patients returning for test-of-cure in Canada found a cefixime MIC threshold of ≥0.12 mg/l to be associated with a treatment-failure rate of 25% (95% CrI 11%–45%) [25]. In 2005, following worrying increases in resistance to the previous therapy, ciprofloxacin, a new recommendation advising that uncomplicated gonorrhoea should be treated with a single dose of cefixime was introduced [26].

**Fig 1:**
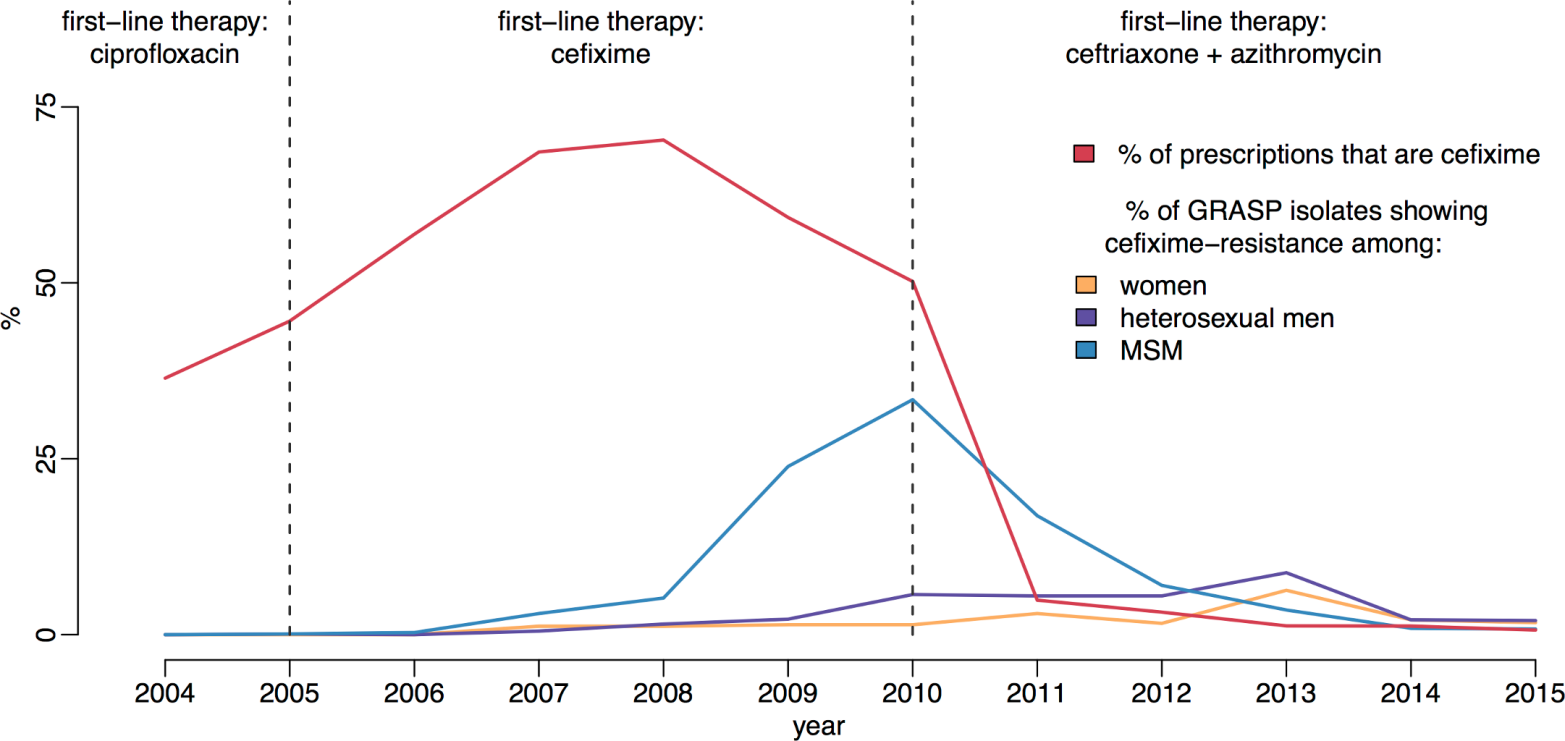
Usage and resistance of cefixime in England and Wales. The proportion of gonococcal isolates in Gonococcal Resistance to Antimicrobials Surveillance Programme (GRASP) that are resistant to cefixime over time is compared with the proportion of gonorrhoea diagnoses treated with cefixime. Dashed lines show the dates of treatment guideline changes. MSM, men who have sex with men.

Fig 1 shows the trends in cefixime prescription and resistance in England. Very little resistance was detected until 2007; however, by 2009 the total level of resistance had passed the 5% threshold at which the WHO recommends that first-line treatment guidelines should be changed [16, 27]. At this time, almost 60% of individuals with gonorrhoea diagnoses were being treated with cefixime [18]. The majority of the resistance was concentrated in the MSM population, where it reached a peak of 33% in 2010 [19]. This evidence, combined with increasingly common reports of cefixime treatment failure, formed the basis for the decision in May 2011 for another update to the treatment guidelines for uncomplicated gonorrhoea [28, 29]. Cefixime was no longer recommended as a first-line treatment and was replaced with a combination of 500 mg ceftriaxone and 1 g azithromycin [4]. Since 2011, cefixime prescribing has fallen drastically, in line with the updated guidelines. Over the same period, the proportion of cefixime-resistant isolates has declined steadily in MSM, falling to less than 1% in 2014 [23].

We hypothesise that the resistance trend observed can be explained by a net fitness benefit to cefixime resistance when cefixime is widely prescribed but a net fitness cost when cefixime prescriptions decline. Understanding the relationship between antibiotic use and the emergence of resistance in gonorrhoea has been identified as a key research agenda [30]. Here our main aim is to further the understanding of the evolutionary dynamics of cefixime resistance and to use this newfound knowledge to inform public health practice. There is still much we do not understand about the natural history of gonorrhoea, especially since unobserved asymptomatic infections have long been thought to be an important reservoir of infection. The proportion of incident cases that are asymptomatic at each bodily site of infection is known to vary but has not been definitively measured [31–33]. Furthermore, the expected duration of carriage of asymptomatic gonococcal infection is not well studied. Estimates have been traditionally in the region of 6 months; however, recent work using genomic data on pairs of known sexual contacts has suggested that a longer duration of carriage can occasionally happen [34]. We therefore developed and applied a Bayesian statistical approach to account for these uncertainties in the epidemiology of gonorrhoea. The analysis was restricted to MSM, the population in which the cefixime-resistant outbreak of gonorrhoea was concentrated.

## Materials and methods

### Epidemiological data

The total number of diagnoses of gonorrhoea in MSM in England between 2008 and 2015 was extracted from the Genitourinary Medicine Clinic Activity Dataset (GUMCAD) [35]. This mandatory reporting system provides data on diagnoses of sexually transmitted infections from sexual health services in England, and the GUMCAD data are published annually by PHE. This yearly number of gonorrhoea diagnoses is denoted *Y*(*t*).

The number of cases of gonorrhoea in MSM that were cefixime resistant and reported by GRASP between 2008 and 2015 were extracted from the corresponding GRASP reports [17–24] and denoted *Y*^res^(*t*). The coverage of GRASP was calculated for every year between 2008 and 2015 by taking the ratio between the number of cases included in GRASP (irrespective of resistance) and the number of GUMCAD diagnoses in the same year. This GRASP coverage proportion is denoted *q*(*t*). GRASP includes a small number of isolates from non-GUM settings, which are not included in GUMCAD. These isolates constitute <3% of the total GRASP sample and are predominantly from women, so while the GRASP data are not strictly a subset of GUMCAD, the effect of the non-GUMCAD cases on the analysis is minimal. The proportion of gonorrhoea cases that were treated with cefixime, as opposed to other antibiotics, was also extracted from the GRASP reports between 2008 and 2015. This time-dependent proportion is denoted *π*(*t*) and illustrated in Fig 1.

A recent estimate based on HIV diagnoses and the European MSM Internet Survey (EMIS) has suggested a United Kingdom MSM population of 3.4% (0.6 million) [36]. This is consistent with the third National Survey of Sexual Attitudes and Lifestyles (Natsal) in which 8.4% of men reported same-sex experience at least once, with 2.6% of men having had a same-sex partner in the last 5 years, putting a plausible range for the MSM population at 0.5 and 1.7 million based on a sexually active male population of 20 million [37, 38]. Therefore, we adopt an estimate of the MSM population size of *N* = 0.6 million.

Given the low prevalence of gonococcal infection in the population, the total population size is not expected to excessively affect the results.

### Transmission model

**Fig 2:**
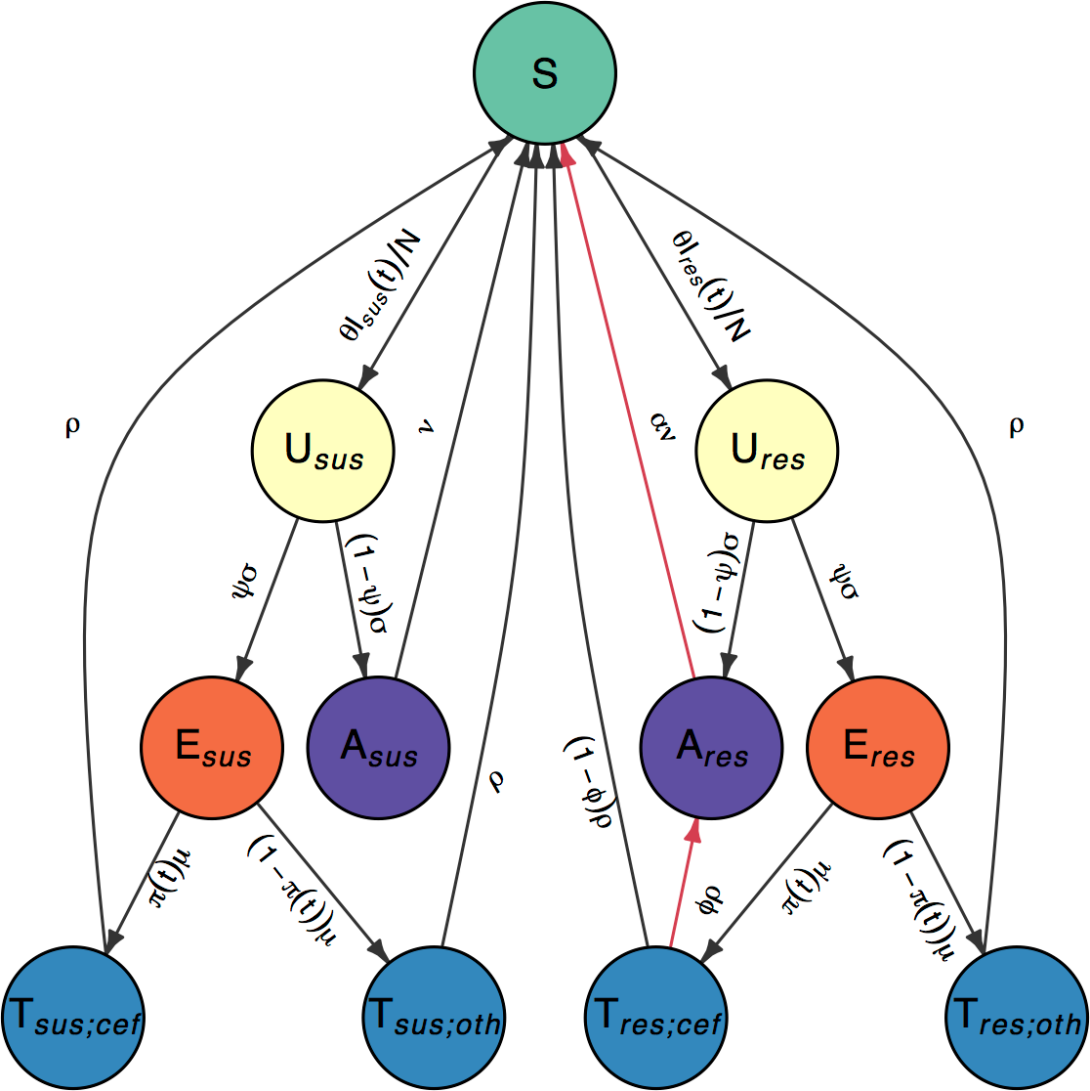
Flow diagram of model compartments with rates of transition between infection states. Susceptible individuals (*S*) become infected with either cefixime-susceptible (*s* = sus) or cefixime-resistant strains (*s* = res). Infections initially pass through an incubation period (*U*_*s*_) before the individuals with the infection either develop symptoms (*E*_*s*_) or remain asymptomatic carriers (*A*_*s*_). Symptomatic individuals seek treatment (*T*_*s*;*p*_) and are prescribed either cefixime (*p* = cef) or another antibiotic (*p* = oth). The 2 sides are symmetric with the exception of the 2 arrows highlighted in red, which correspond to the cost and the benefit of resistance.

In order to investigate the fitness cost and benefit of cefixime resistance in gonorrhoea, we created a stochastic compartmental model, illustrated in Fig 2 with notation summarised in Table 1. It was important to use a stochastic model because of the small number of resistant cases detected by GRASP in the early and late stages of the outbreak that would not be captured by a deterministic model. High rates of reinfection with gonorrhoea have been observed, suggesting low levels of acquired immunity [39], and experimental urethral infection in male volunteers found no protection was conferred on repeat infection with an identical strain 6 months after the initial infection [40]. It was therefore assumed that no immunity was conferred upon recovery from infection. A closed population of size *N* was assumed because of the short time period under consideration. Individuals are initially susceptible (*S*). They become infected with strain *s* ∈ {sus,res}, denoting cefixime-susceptible and cefixime-resistant strains, respectively. The model assumes that strains do not vary in transmissibility and that the rate of infection from an infectious individual to a susceptible individual is *θ*/*N*. Infected individuals initially pass through an incubation period (*U*_*s*_), which they leave at rate *σ*. A proportion *ψ* of those infected then go on to develop symptoms (*E*_*s*_), whereas the remainder enter an asymptomatic stage (*A*_*s*_). Gonococcal infection can occur in the rectum, pharynx, and/or urethra, resulting in different rates of onward transmission and probabilities of developing symptoms [41]. We do not explicitly model separate sites of infection; therefore, the rate of transmission, *θ*, and the likelihood of developing symptoms, *ψ*, should be seen as an average for any infection site. Recovery from asymptomatic infection happens (either naturally or following unrelated antibiotic treatment) at rate *ν* for the susceptible strain and at rate *αν* for the resistant strain. The parameter *α* therefore represents the fitness cost of cefixime resistance. The infected population for each strain *s* is denoted *I*_*s*_ = *U*_*s*_ + *E*_*s*_ + *A*_*s*_, and the total infected population is denoted *I* = *I*_sus_ + *I*_res_. All infected individuals are assumed to be infectious. The symptomatic individuals (*E*_*s*_) seek treatment at rate *μ*. A time-varying proportion *π*(*t*) are treated with cefixime (*T*_*s*;cef_), whereas the remaining 1−*π*(*t*) are treated with other antibiotics (*T*_*s*;oth_). The treated individuals recover from the infection and become susceptible again at rate *ρ*, with the exception of a proportion *ϕ* of the individuals infected with a cefixime-resistant strain who have been treated with cefixime (*T*_res;cef_) but experience treatment failure and become asymptomatically infected (*A*_res_) [42, 43].

**Table 1:** Parameter notations and prior and posterior distributions.

	Parameter description	Unit	Prior distribution	Prior mean (95% CrI)	Posterior mean (95% CrI)
*A*_sus_(0)	Initial carriage of cefixime-susceptible infection	#	Uniform(0,∞)	n/a	618 (411–819)
*A*_res_(0)	Initial carriage of cefixime-resistant infection	#	Uniform(0,∞)	n/a	49 (20–92)
*θ*	Rate of transmission per partner	per year	Gamma(44,10)	4.4 (3.2–5.8)	5.2 (4.1–6.5)
*ψ*	Infections that become symptomatic	%	Uniform(0,1)	50 (2.5–97.5)	63 (48–76)
*σ*	Rate of departing incubation period	per year	Gamma(17,0.22)	77 (45–118)	77 (46–119)
*ν*	Rate of recovery from asymptomatic infection	per year	Gamma(8,3.45)	2.3 (1.0–4.2)	1.8 (0.9–3.0)
*μ*	Rate of seeking treatment when symptomatic	per year	Gamma(3,0.02)	150 (31–361)	136 (33–350)
*ρ*	Rate of recovery following treatment	per year	Gamma(101,1.9)	53 (43–64)	53 (43–63)
*α*	Increased recovery from cefixime-resistant infection	n/a	Uniform(0,∞)	n/a	1.8 (1.4–2.4)
*ϕ*	Treatment failure rate for cefixime-resistant infections treated with cefixime	%	Uniform(0,1)	50 (2.5–97.5)	83 (53–99)

### Calculation of the basic reproduction number

The basic reproduction number, *R*_0_, is a measure of the reproductive capacity of an infectious agent and is defined as the average number of secondary cases of gonorrhoea arising from the introduction of a typical infected individual in a completely susceptible population. Where there is direct competition between strains, as in the situation we are modelling, the strain with the highest *R*_0_ will outcompete the others.

To calculate *R*_0_, we must consider the generation time, defined as the expected time from an individual becoming infected to infecting another individual. By considering the expected time spent in each compartment of the model corresponding to infection with the susceptible strain (i.e., states *U*_sus_, *E*_sus_, and *A*_sus_ in Fig 2), we derive an analytical expression of the basic reproduction number $R_{0}^{\mathrm{sus}}$ for the susceptible strains using the next-generation method, as detailed in S2 Appendix [44]:

$$R_{0}^{\mathrm{sus}}=\theta (\frac{1}{\sigma }+\frac{1-\psi }{\nu }+\frac{\psi }{\mu })$$ (1)

Similarly, the basic reproduction number $R_{0}^{\mathrm{res}}$ for the resistant strains is given by the following:

$$R_{0}^{\mathrm{res}}=\theta (\frac{1}{\sigma }+\frac{1-\psi +\phi \pi \psi }{\alpha \nu }+\frac{\psi }{\mu })$$ (2)

Therefore, by equating Eqs 1 and 2, we can obtain the level of cefixime prescriptions above which the resistant strains become fitter than the cefixime-susceptible strains:

$$\pi^{\left\{R_{0}^{\mathrm{sus}}=R_{0}^{\mathrm{res}}\right\}}=\frac{\left(1-\psi )(\alpha -1\right)}{\phi \psi }$$ (3)

### Bayesian inference

We considered the data as a partially observed Markov process, with the number of GUMCAD recorded cases, *Y*(*t*), and GRASP reported resistant cases, *Y*^res^(*t*), being the observed realisations of the underlying unobserved processes: the total incidence of gonorrhoea infections, *Z*(*t*), and the incidence of cefixime-resistant infections, *Z*^res^(*t*). The reporting process assumed that 90% of all gonorrhoea diagnoses are recorded by GUMCAD with a 10% margin of error, consistent with findings that 6%–9% of gonorrhoea is diagnosed in a GP setting [45].

Y(t)∼Normal(0.9Z(t),0.05Z(t))

The probability of a cefixime-resistant case of gonorrhoea being sampled by the GRASP study was assumed to be Poisson distributed with a sampling probability denoted *q*(*t*) derived from the coverage of the GRASP study over 2008 to 2014:

$$Y^{\mathrm{res}}(t)∼\mathrm{Poisson}(q(t)Z^{\mathrm{res}}(t))$$

Based on these observations, we aimed to infer the values of the 10 parameters: *A*_sus_(0),*A*_res_(0),*θ*,*ψ*,*σ*,*ν*,*α*,*μ*,*ρ*, and *ϕ*.

An analytical expression for the likelihood of the observed data given our model is not available, so we obtained an unbiased estimate of the likelihood using a particle filter [46]. The estimated likelihood was then incorporated into a particle Monte Carlo Markov Chain (pMCMC), which was used to obtain a sample from the posterior distribution of the model parameters [47]. The difficulty of exploring the posterior parameter space with the pMCMC algorithm increases with the number of parameters. The number of symptomatic cases was therefore initialised as *E*_sus_(0) = *E*_res_(0) = 0, as after a few days of simulation, these variables reached the stochastic equilibrium values implied by the model and the parameters.

The model fitting was implemented using the R package pomp, which includes a pMCMC algorithm that can be used to perform Bayesian inference [48]. The algorithm was modified to enable parallel computation. The particle filter estimation of the likelihood was based on 1,000 particles, which was sufficiently robust to estimate the likelihood. The pMCMC was run for 1.1 million iterations, with the first 10% discarded as burn-in and the remainder thinned by a factor of 100. Four separate chains were run with dispersed starting points and compared using the R package coda [49]. The chains appeared to have converged to the same posterior distribution based on the multivariate version of the Gelman-Rubin diagnostic, which was less than 1.1 for all inferred parameters [50, 51]. To ensure maximum robustness, the samples from the 4 chains were then combined and found to have an effective sample size of more than 200 for all parameters.

### Prior distributions of parameters

Bayesian inference requires setting plausible priors for the model parameters. We used highly uninformative Uniform(0,1) priors for the 2 proportion parameters *ϕ* and *ψ* and Uniform(0, ∞) priors for the 3 parameters *A*_sus_(0),*A*_res_(0), and *α*, which is an improper distribution but does not lead to an improper posterior distribution. For the 5 remaining parameters *θ*,*ν*,*σ*,*μ*, and *ρ* we assigned informative Gamma priors based on a literature review, as summarised in Table 1.

The transmission rate of infection, represented by the parameter *θ*, encompasses both the average number of sexual partners per year and the transmission probability per partnership. The Natsal-3 survey observed a mean number of sexual partners per year for MSM of 4.4 [37], and we would therefore expect *θ* to be slightly lower, to reflect the fact that not all contacts result in transmission. The prior distribution for *θ* was therefore set such that it was between 2.9 and 6.3 with 99% prior weight.

The expected duration of carriage for asymptomatic gonorrhoea is not well measured. A study of 18 asymptomatic infected men saw no resolution in urethral infection in the 165 days before they received treatment [43]. Estimates of the duration of carriage in modelling studies have been based on calculations that take into account observed prevalence and an assumed proportion of unobserved infection and often assume an average of 6 months [52–54]. This is confirmed by recent work using genomic data, in which the greatest observed time to most recent common ancestor for bacterial genomes from known contact pairs was 8 months [34]. The duration of carriage may depend on the infection site; for pharyngeal gonorrhoea, it has been estimated to be 12 weeks, and for rectal infection, it has been estimated to be 1 year [55, 56]. The parameter *ν* was therefore assigned a prior that corresponded to a mean duration of carriage between 3 months and 1 year with 99% prior weight.

The duration of the incubation, symptomatic, and treatment stages of infection have been estimated to be short, in the region of days rather than weeks [57–59]. Gamma priors were accordingly assigned to each of the 3 parameters *σ*,*μ*, and *ρ*.

## Results

### Estimation of model parameters

**Fig 3:**
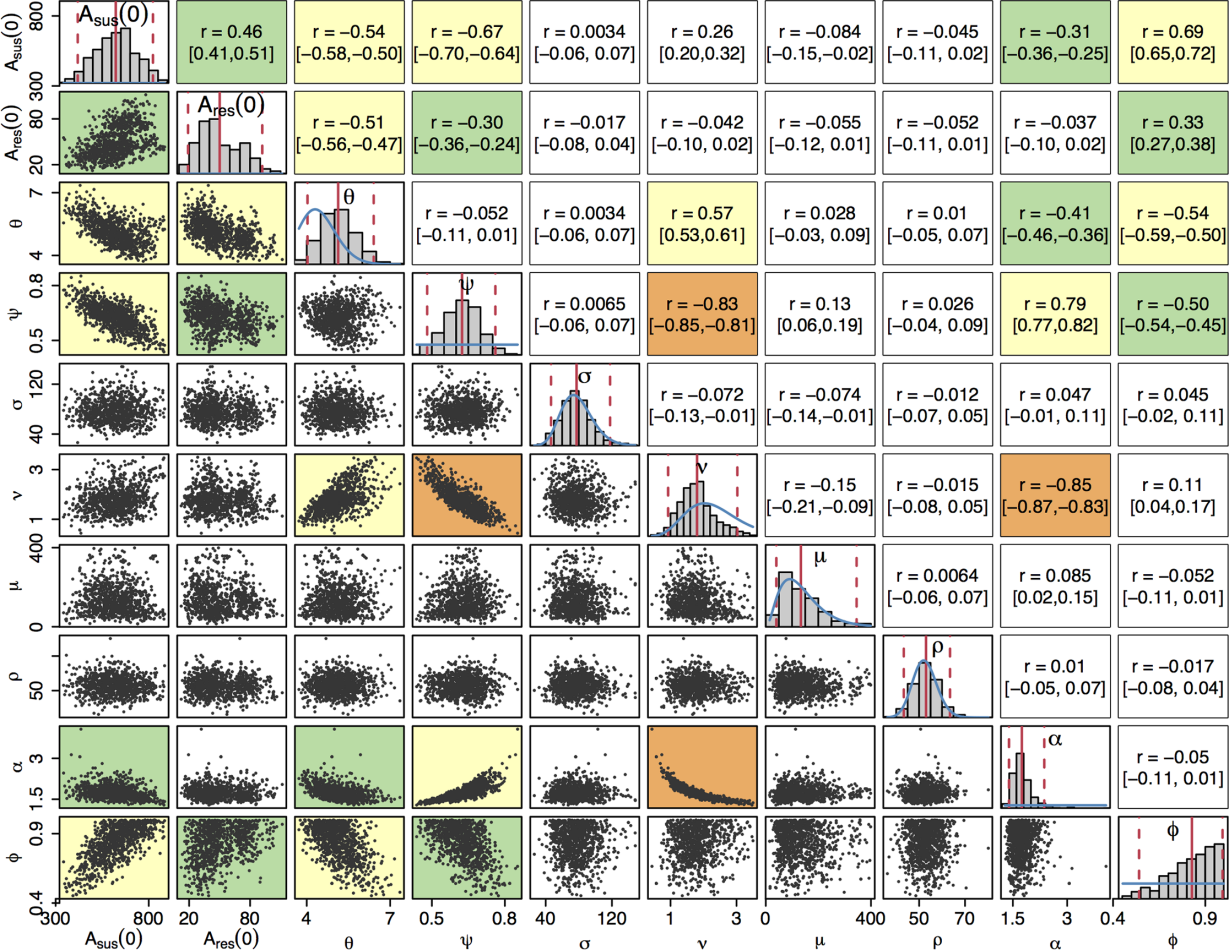
Posterior distributions of parameters. The diagonal plots show histograms of the posterior distributions for all sampled parameters. The blue lines show prior distributions, and the red lines indicate posterior mean and 95% credible intervals. The plots below the diagonal show scatter plots based on 1,000 samples from the posterior, illustrating the relationships between pairs of estimated parameters. An orange background indicates a correlation higher than 0.8, a yellow background indicates a correlation between 0.5 and 0.8, a green background indicates a correlation between 0.3 and 0.5, and a white background indicates a correlation less than 0.3. The plots above the diagonal show the corresponding correlation coefficients with the 95% credible intervals in parentheses.

We fitted our model of gonorrhoea transmission to 2 different time series over the years 2008 to 2014: the total number of gonorrhoea diagnoses in MSM in England [60] and the incidence of cefixime-resistant gonorrhoea [17–23]. The posterior distribution of parameters shown in Fig 3 was obtained through Bayesian inference, implemented using a pMCMC method [47]. For each parameter, we report the posterior mean estimate and 95% credible interval shown in brackets (Table 1). The model suggests that at the end of 2007, the first year that cefixime-resistant cases were detected by GRASP [16], there were 618 cases (95% CrI 411–819) of asymptomatic cefixime-susceptible gonorrhoea (*A*_sus_(0)) and 49 cases (95% CrI 20–92) of asymptomatic cefixime-resistant gonorrhoea (*A*_res_(0)).

The posterior distribution of the rate of transmission, *θ*, suggests a higher mean rate of infection than the prior expectation: 5.2 (95% CrI 4.1–6.5), but the prior and posterior credible intervals overlap to a large extent, suggesting that the results are consistent with our prior knowledge. Our model predicts that the proportion *ψ* of infections that become symptomatic is 63% (95% CrI 48%–76%). The 3 parameters *σ*, *μ*, and *ρ*, corresponding respectively to the durations of the incubation period, the symptomatic infection before seeking treatment, and the treatment phase, had posterior distributions similar to their prior distributions, indicating that the prior distributions were appropriate but that there is little additional information on these parameters in the data set. The posterior distribution of *ν* has a slightly lower mean than the prior distribution, implying a longer mean duration of carriage of 203 days (95% CrI 122–397). The prior and posterior credible intervals still intersect to a large extent so there is not significant evidence of a departure from the prior based on the data.

The last 2 parameters, *α* and *ϕ*, capture the difference between the susceptible and resistant strains in our model. The model predicts that in order to replicate observed incidence patterns, recovery from asymptomatic cefixime-resistant gonorrhoea occurs *α* = 1.8 (95% CrI 1.4–2.4) times faster than recovery from asymptomatic cefixime-susceptible gonorrhoea, giving rise to a fitness cost. The model suggests a treatment failure proportion of *ϕ* = 83% (95% CrI 53%–99%) for resistant gonorrhoea treated with cefixime such that resistance confers a fitness benefit in an environment in which cefixime is highly prescribed.

Beyond the marginal posterior distributions of the parameters described above, it is informative to study their posterior correlations. The pairwise posterior relationships between the 10 parameters are depicted in Fig 3. Parameters *σ*, *μ*, and *ρ* did not show a strong correlation with any parameters; as expected, the short duration of the incubation, symptomatic, and treatment stages of infection led to these parameters contributing relatively little to the dynamics of infection. The strongest correlation was found between *ν* and *α*, *r* = −0.85 (95% CrI −0.87 to −0.83), corresponding to the trade-off required to maintain the duration of carriage of resistant infection, which is equal to 1/(*αν*) and accounts for the nonlinearity of the relationship. Parameters *ν* and *ψ* were also highly negatively correlated, *r* = −0.83 (95% CrI −0.85 to −0.81), which corresponds to the trade-off between duration of carriage, 1/*ν*, and the proportion of infections entering the carriage state, (1−*ψ*). The 2 negative correlations of both *α* and *ψ* with *ν* lead to a positive correlation between *α* and *ψ*.

### Posterior predictive analysis

**Fig 4:**
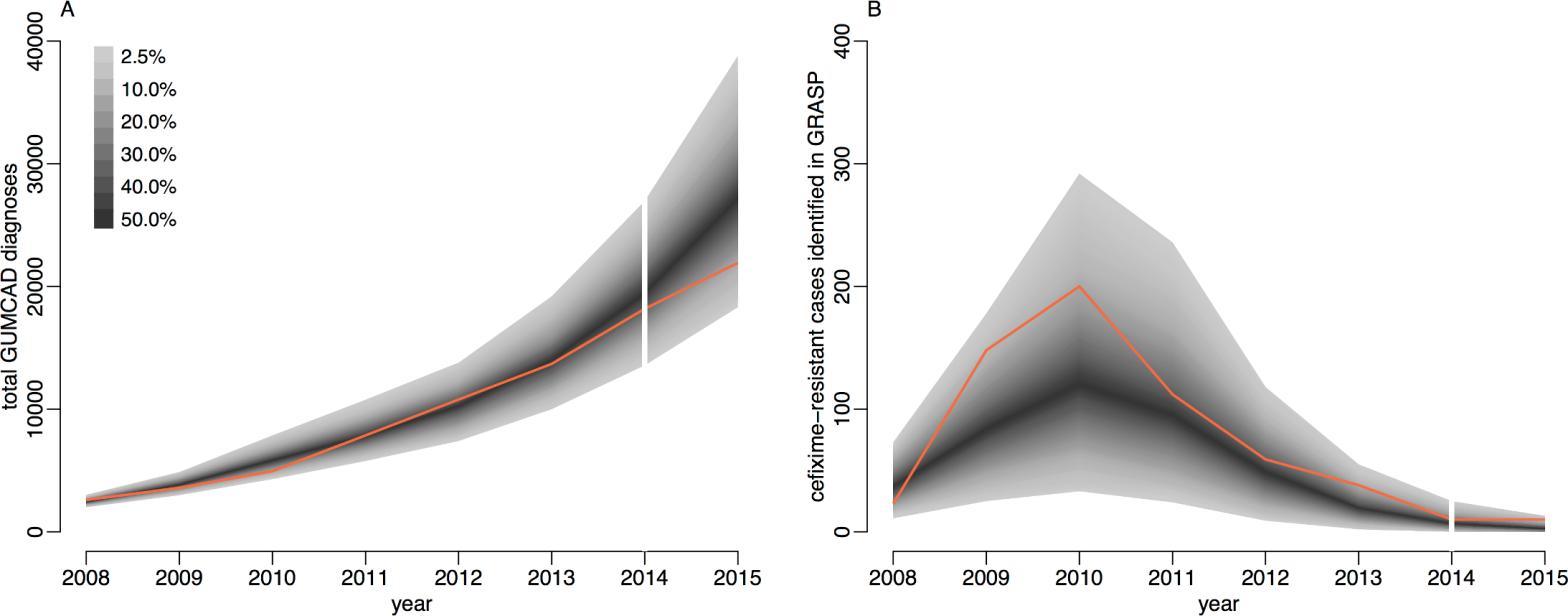
Comparison of simulated and observed cases of gonorrhoea. Panel A shows the total number of cases, and panel B shows only the cefixime-resistant cases. Observed data are shown in orange, with the shaded area showing the 95% posterior predictive interval (based on 1,000 simulations using samples from posterior distribution). Note different scales. GUMCAD, Genitourinary Medicine Clinic Activity Dataset.

The total number of gonorrhoea cases in MSM observed by GUMCAD and the number of cefixime-resistant infections isolated in MSM by GRASP were compared with simulated data sets using parameters sampled from their posterior distributions to assess the goodness of fit of our model to the data. One thousand parameter sets were sampled, and 1 simulation was performed using each set. Fig 4 demonstrates that the simulated data closely emulate the real data. The real data are within the 95% predictive intervals at all time points, indicating a good fit of the model to the data [61].

The total number of cases of gonorrhoea observed by GUMCAD and the number of cefixime-resistant cases observed by GRASP in 2015 [24] were not used in the model-fitting process and were used to provide an independent check of the model fit. Both data points are within the 95% probability intervals predicted by our model: 21,915 gonorrhoea diagnoses were recorded by GUMCAD, compared to 27,475 (95% CrI 18,307–38,810) diagnoses predicted by the model; 10 cefixime-resistant cases were recorded by GRASP, compared to 4 (95% CrI 0–13) cases predicted by the model. Our modelling suggests that in 2015, 1.7% (95% CrI 1.0%–2.6%) of MSM in England may have been carriers of asymptomatic gonorrhoea, and 0.2% (95% CrI 0.1%–0.4%) may have had symptomatic gonorrhoea, with an overall prevalence of 2.0% (95% CrI 1.1%–2.9%). Our results suggest that asymptomatic gonorrhoea accounted for 89% (95% CrI 82%–93%) of onward transmission in MSM.

### Comparative analysis of basic reproduction numbers

**Fig 5:**
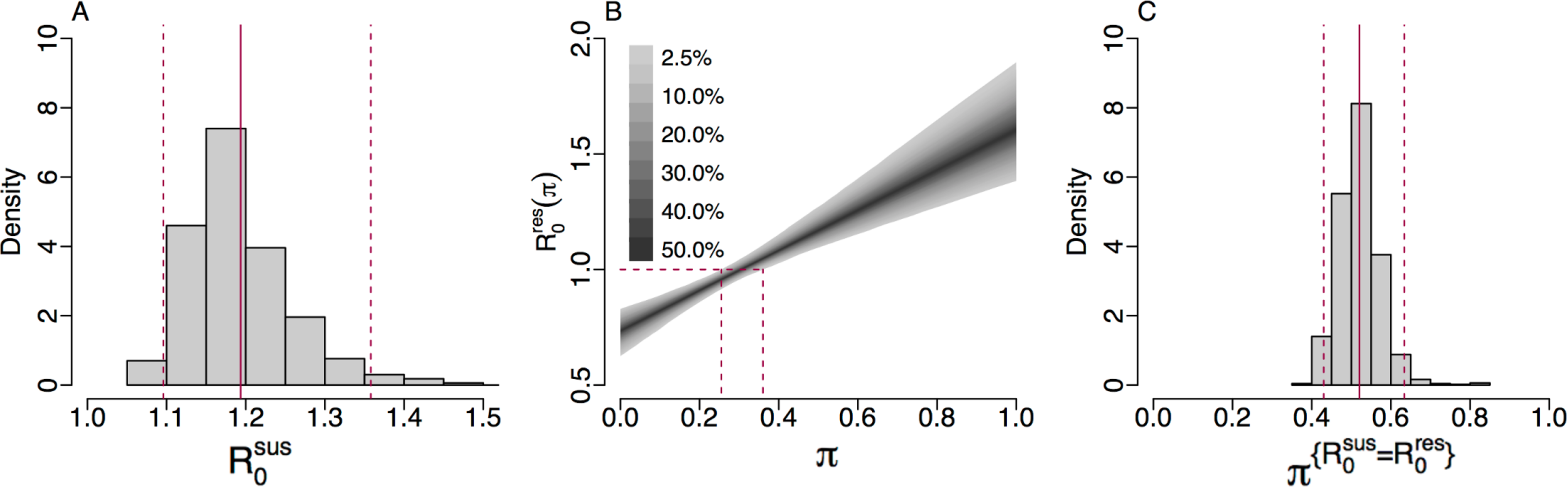
(A) Histogram of the posterior estimate of $R_{0}^{\mathrm{sus}}$. (B) The 95% credible interval of $R_{0}^{\mathrm{res}}(\pi )$ against *π* with dashed lines showing the 95% credible interval for $\pi^{\left\{R_{0}^{\mathrm{res}}=1\right\}}$. (C) Histogram of the posterior estimate of $\pi^{\left\{R_{0}^{\mathrm{sus}}=R_{0}^{\mathrm{res}}\right\}}$: the threshold of cefixime prescriptions above which $R_{0}^{\mathrm{res}}>R_{0}^{\mathrm{sus}}$.

A key threshold in epidemic theory associates the persistence of disease in a population with a basic reproduction number greater than 1 [62]. Using Eq 1, we obtain a posterior estimate for the basic reproduction number for cefixime-susceptible infection of $R_{0}^{\mathrm{sus}}$ = 1.19 (95% CrI 1.10–1.36), which suggests that the cefixime-susceptible strain of gonorrhoea is expected to persist in the population without further intervention (Fig 5A). Under our hypothesis, the basic reproduction number for cefixime-resistant gonorrhoea depends on the frequency of cefixime prescription (Eq 2) and thus can be considered as a function $R_{0}^{\mathrm{res}}(\pi )$ where *π* is the proportion of gonorrhoea diagnoses being treated by cefixime. In the 2 extreme cases when no cefixime is prescribed (*π* = 0, meaning that the treatment is always effective) and only cefixime is prescribed (*π* = 1, meaning that only a proportion 1−*ϕ* of the treatment is effective), we estimate a basic reproduction number for resistant gonorrhoea of $R_{0}^{\mathrm{res}}(0)$ = 0.73 (95% CrI 0.63–0.83) and $R_{0}^{\mathrm{res}}(1)$ = 1.61 (95% CrI 1.38–1.90), respectively. At its height in 2008, the frequency of cefixime prescriptions was 70%, and we estimate that at this time the basic reproduction number for resistant gonorrhoea was $R_{0}^{\mathrm{res}}(0.7)$ = 1.35 (95% CrI 1.21–1.52). The former estimate is ≤1, whereas the latter 2 are ≥1; this is consistent with the fact that between 2005 and 2010, when cefixime was often used to treat gonorrhoea, resistance to cefixime increased, whereas with the discontinuation of cefixime usage from 2011, resistance has decreased.

We can estimate the frequency of cefixime prescriptions above which we expect the resistant strain to persist, corresponding to when the fitness benefit of cefixime resistance is greater that its fitness cost, by setting $R_{0}^{\mathrm{res}}(\pi )=1$ and solving for *π* in Eq 2. We denote this threshold $\pi^{\left\{R_{0}^{\mathrm{res}}=1\right\}}$ and thus obtain a posterior estimate of $\pi^{\left\{R_{0}^{\mathrm{res}}=1\right\}}$ = 0.31 (95% CrI 0.26–0.36) (Fig 5B). This result suggests that up to a quarter of gonorrhoea treatments could be with cefixime monotherapy without causing a cefixime-resistant epidemic. Another important threshold is the level of cefixime prescriptions above which the resistant strain of gonorrhoea is fitter than the susceptible strain. We denote this threshold $\pi^{\left\{R_{0}^{\mathrm{sus}}=R_{0}^{\mathrm{res}}\right\}}$. By setting $R_{0}^{\mathrm{res}}=R_{0}^{\mathrm{res}}$ and equating Eqs 1 and 2, we obtain a posterior estimate of $\pi^{\left\{R_{0}^{\mathrm{sus}}=R_{0}^{\mathrm{res}}\right\}}$ = 0.55 (95% CrI 0.44–0.66) (Fig 5C).

### Impact of cefixime usage on simulated resistance trends

**Fig 6:**
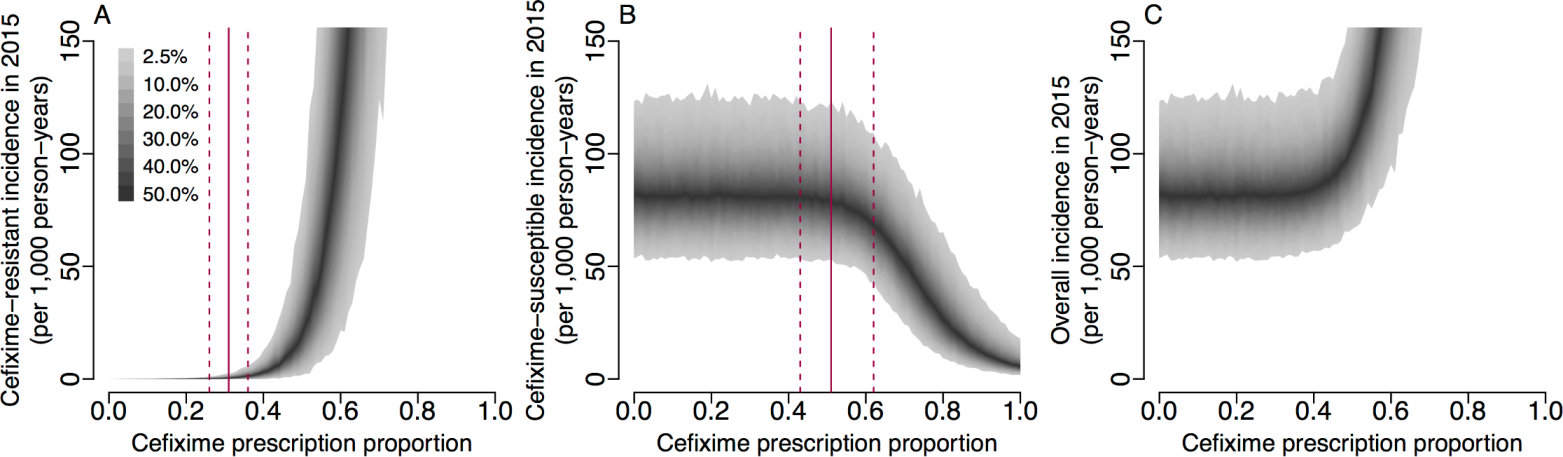
Incidence of gonorrhoea in 2015 based on simulations from 2004 to 2015 with varying levels of cefixime prescribing. (A) Incidence of the cefixime-resistant strain. The red lines show the 95% credible interval for $\pi^{\left\{R_{0}^{\mathrm{res}}=1\right\}}$. (B) Incidence of the cefixime-susceptible strain. The red lines show the 95% credible interval for $\pi^{\left\{R_{0}^{\mathrm{sus}}=R_{0}^{\mathrm{res}}\right\}}$. (C) The overall incidence from both cefixime-resistant and cefixime-susceptible strains. The shaded areas show the 95% posterior predictive intervals (based on 1,000 simulations using samples from posterior distribution).

The basic reproduction numbers derived above are informative but do not capture completely the complex dynamics of infection transmission that occur when accounting for stochasticity, competition between susceptible and resistant strains, and non-negligible fractions of the population becoming infected. To further study the impact of cefixime prescribing on the cefixime-resistant and cefixime-susceptible epidemics, we performed stochastic model simulations over 8 years from 2008 to 2015 using parameters drawn from their posterior distributions and examining scenarios with a frequency of cefixime prescriptions ranging from no use of cefixime (*π* = 0) to all gonorrhoea cases being treated by cefixime (*π* = 1). The prescription frequency in each case was kept constant throughout the entire simulation period. Fig 6A shows that, when fewer than 27% of gonorrhoea cases were treated with cefixime, there was a 95% probability that the resistant outbreak no longer persisted in 2015. This is comparable to our estimate of $\pi^{\left\{R_{0}^{\mathrm{res}}=1\right\}}$ = 0.31 (95% CrI 0.26–0.36) (Fig 5B), the level of prescriptions above which the fitness benefit of cefixime resistance is greater than the fitness cost.

Fig 6B shows that, when more than 50% of gonorrhoea cases were treated with cefixime, the simulated incidence of cefixime-susceptible infection began to fall, with the cefixime-resistant strain becoming more common. This supports our analytical estimate of $\pi^{\left\{R_{0}^{\mathrm{sus}}=R_{0}^{\mathrm{res}}\right\}}$ = 0.55 (95% CrI 0.44–0.66) (Fig 5C), the level of cefixime prescriptions above which the resistant strain becomes fitter than the susceptible strain. If cefixime were used to treat more than 58% of cases, then the level of cefixime resistance would become greater than 50% at the end of the 8-year simulation period, and if cefixime were used to treat all cases, resistance would be close to 100%.

## Discussion

### Main findings

We have used mathematical modelling and Bayesian inference methods to uncover insights into the dynamics of cefixime resistance in gonorrhoea. We quantified both the fitness cost and fitness benefit of resistant strains, which allowed us to make predictions about the future prevalence of resistance as a function of how often cefixime is prescribed. Our results indicate that cefixime could be used to treat uncomplicated cases of gonorrhoea without incurring the risk of causing a resistant epidemic like the one that happened in 2007–2012, provided its frequency of use were controlled, enabling continued use of an ‘abandoned’ antibiotic. Our analysis uses the complementary approaches of analysing the basic reproduction number and posterior predictive analysis to suggest that cefixime could be used to treat up to 25% of cases. This threshold should still be used cautiously, however, for reasons described below. *N*. *gonorrhoeae* has developed resistance to each first-line antibiotic in turn, from penicillin to third-generation cephalosporins. Our modelling work lays the foundation for a rational scientific approach to extending and ultimately preserving the usefulness of existing antibiotics.

### Study strengths and limitations

The strengths of our methodology reside in the explicit stochastic model of gonorrhoea infection we developed and the state-of-the-art approach to Bayesian inference employed to estimate model parameters. We were able to explain and reproduce the observed boom-and-bust trend of cefixime resistance by modelling both the fitness cost and the fitness benefit of resistant strains relative to susceptible strains. These 2 fitness properties had not been estimated before, and yet, we demonstrate that they are the key to making predictions about future resistance and incidence levels and therefore to making rational decisions about antibiotic usage policies. We deliberately kept our model as simple as possible, firstly to avoid the computational challenges that arise with a complex model and secondly to minimise the number of parameters for which the data analysed here would not be informative. However, it is important and interesting to consider the validity of our model assumptions and the effect they could have on our results.

Our model ignores the fact that gonorrhoea can infect different body sites, including in MSM the rectum, pharynx, or urethra, which results in different rates of onward transmission and probability of developing symptoms [41]. Reliable statistics are lacking for the relative prevalence, transmissibility, and pathogenicity of gonorrhoea by site of infection; consequently, this aspect would be difficult to add to our model. The parameters we estimate should be seen as averages for any infection site. Unless sexual practices were to change over time, we would expect the relative contributions to prevalence, symptomatic infections, asymptomatic infections, and transmission of each anatomical site to remain the same over time. We have no evidence that sexual practices did change over the time period considered; therefore, we expect that this averaging should have a minimal effect on the overall results. Likewise, we did not model heterogeneity in sexual behaviours or the underlying sexual network within the population, both of which can affect gonorrhoea spread within a population [63, 64], but these would be expected to impact both susceptible and resistant strains in the same fashion and therefore not affect our results on the dynamics of cefixime resistance. Indeed, a recent modelling study showed that differences of behaviour do not explain differences in resistance levels [65]. Our model assumes that treating a cefixime-resistant infection with cefixime results in treatment failure with probability *ϕ*, whereupon individuals become asymptomatic carriers. This is necessary to provide a significant benefit to the resistant strain, allowing it to spread when cefixime was used frequently. Some treatment failures would in fact have been detected and led to successful re-treatment, which we do not model explicitly but can be thought of as being part of the probability 1−*ϕ* of treatment success in spite of using cefixime for a resistant case. Control measures such as repeated testing of high-risk individuals or partner notifications are also not modelled explicitly but would likely affect the prevalence of both susceptible and resistant cases in a similar fashion and therefore not alter significantly our conclusions about the fitness differences caused by resistance. Furthermore, partner notification often occurs at low rates: only circa 10% of gonorrhoea that is diagnosed in England and reported through GUMCAD is found through partner notification, with the proportion likely to be lower in MSM than among heterosexuals [60].

It was assumed that all cefixime-susceptible infections were cured, regardless of which antibiotic was prescribed. The prescription data show that between 2008 and 2015 the vast majority of non-cefixime prescriptions were for ceftriaxone, either alone or in combination with azithromycin, so the assumption of cure is reasonable given that ceftriaxone resistance reports remain sporadic in England. The GRASP data we used in this study reflect a definition of cefixime resistance (MIC ≥ 0.125 mg/l) that is less stringent than the European Committee on Antimicrobial Susceptibility Testing (EUCAST) definition (MIC > 0.125 mg/l) and do not include information about concurrent prescription of azithromycin against any concomitant *Chlamydia trachomatis* infection [66], even though this is also an effective treatment against gonorrhoea [67]. Both these factors should contribute to a relatively low probability *ϕ* of treatment failure. Our estimate of *ϕ* was 83% with a wide 95% credible interval from 53% to 99%. A study on treatment failure in Toronto, Canada, estimated that only 25% (95% CrI 11%–45%) of patients with a cefixime MIC of >0.12 mg/l experienced treatment failure when treated with cefixime [25]. This figure is based on 7 out of 28 patients who returned for test of cure. A further 31 patients with a cefixime MIC of >0.12 mg/l detected at first treatment did not return for test of cure, resulting in the low sample size. The study reports that, initially, 13 patients with a cefixime MIC of >0.12mg/l failed the test of cure; however, 4 of these did not have an explicit denial of sexual re-exposure in their medical record. The study makes the strong assumption that all of these cases were in fact reinfections, which may have artificially reduced the treatment failure rate observed.

Our model implicitly assumes that there is no coinfection with both strains and no evolution of resistance happening within host. Ignoring within-host competition between resistant and susceptible strains following coinfection is justified here by the fact that both strains have low prevalence, making coinfection very unlikely. In a genomic study in the United States, only 2 clades of cefixime resistance were detected, suggesting that de novo acquisition of resistance is rare [3]. This simplification has been used in a number of other studies on the epidemiology of antimicrobial resistance [68, 69]. Within-host evolution of resistance was included in a recent gonorrhoea modelling study [65], but clearly, this is a rare event that only increases by 1 the number of resistant infections, which we estimated to be 49 at the start of the epidemic simulation on 1 January 2008.

Finally, our model also makes assumptions concerning the cost of cefixime resistance. The fitness cost of the mutation conferring resistance is assumed to be constant over time; however, compensatory mutations that reduce the initially high fitness cost of antibiotic resistance have been observed in other bacterial pathogens [70, 71]. It is clear from our analysis that there was a substantial fitness cost to cefixime resistance when the prescription protocol was changed in 2010, which is the reason why the resistance level subsequently fell. We cannot rule out that compensatory mutations took place after resistance initially emerged, but this would mean that the initial cost was even higher, and in these conditions, resistance would have been unlikely to emerge at all. Our formulation of the dynamics of the fitness cost of resistance was via a reduction in the duration of asymptomatic carriage. In the absence of evidence of the resistance mechanism, the fitness cost could plausibly be modelled through reduced transmissibility of the resistant strain [65]; we have confirmed with analysis that this would not affect our overall conclusions, in particular regarding the basic reproduction number analysis and predictions of the impact of cefixime usage on future resistance trends.

### Implications for policy and research

The ceftriaxone-azithromycin dual therapy is currently effective, but it represents a last resort; thus, we urgently need a strategy for what would be done if it stopped working. It is likely to be just a matter of time before this happens, with the first reported failure of the dual therapy having occurred in 2015 [72]. Resistance to azithromycin was detected in a recent outbreak that started in the north of England [7] and is now reported in almost 10% of tested isolates [24]. Resistance to ceftriaxone remains rare, but MIC levels have been steadily increasing [73]. If alternative treatment options could be used, even for a minority of cases, then it would delay and maybe even prevent the emergence of resistance to the dual-therapy antibiotics by reducing the fitness benefit it would confer.

For some previously used antibiotics, such as penicillin or ciprofloxacin, significant levels of resistance remain in the gonococcal population (24% and 39% in 2015, respectively [24]) such that they cannot be recommended even for a small fraction of cases. These antibiotics could be prescribed only if drug sensitivity could be quickly established, for example, using real-time PCR assays [74, 75], or whole genome sequencing [76, 77], which both remain experimental. In contrast, the fact that resistance to cefixime has become very low in England (around 1% in 2015, [24]) makes it a prime candidate for return into action without the need for case-by-case susceptibility testing. Using 2 different methods—namely, a basic reproduction number analysis and posterior predictive simulations—we estimated that a quarter of gonorrhoea cases could be treated with cefixime. This model prediction is based on a randomised treatment strategy at the patient level whereby cefixime is allocated to a subset of uncomplicated cases for which resistance profiles are unknown. Combining this treatment strategy with point-of-care susceptibility testing could bring further benefits to avoid prescribing antibiotics for which resistance is detected. When coinfection with chlamydia is demonstrated or suspected, cefixime could be combined with azithromycin; this or other dual therapies could be useful more generally as well, and future modelling work should consider the evolutionary dynamics of multiple antibiotics concurrently. Perhaps the greatest threat posed by this proposed strategy would be the evolution of compensatory mutations that could reduce the fitness cost of cefixime resistance. As previously mentioned, compensatory mutations do not seem to have emerged during the 2007–2012 cefixime-resistant epidemic, but if they did occur, then the acceptable prescribing proportion would be lowered, and the probability of persistence of cefixime resistance increased. Therefore, a redeployment of cefixime would require the continuation, and perhaps reinforcement, of monitoring of resistance trends in England [8] and beyond [78, 79]. Ideally, surveillance systems would routinely record the prescription and treatment outcome for each individual case, as part of antimicrobial stewardship and monitoring of antibiotic resistance.

### Conclusion

Fighting antibiotic-resistant gonorrhoea requires initially understanding the dynamics of how resistance emerges and spreads, in order to make informed decisions about treatment. Here we focused on an antibiotic, cefixime, which was previously used but had to be abandoned due to rising resistance levels. We estimated that resistance to cefixime comes at a significant cost for the pathogen; thus, when cefixime is not used, resistance tends to disappear. We also quantified the benefit of cefixime resistance for gonorrhoea, which is an increasing function of how often cefixime is used to treat gonorrhoea. Our results suggest that cefixime could be used again to treat a minority of gonorrhoea cases without risk of worsening the resistance problem.

## Supporting information

S1 Appendix Model equations and stochastic simulations. (PDF)

S2 Appendix Derivation of basic reproduction numbers. (PDF)

## Abbreviations

EUCAST: European Committee on Antimicrobial Susceptibility Testing
GRASP: Gonococcal Resistance to Antimicrobials Surveillance Programme
GUMCAD: Genitourinary Medicine Clinic Activity Dataset
MIC: minimum inhibitory concentration
MSM: men who have sex with men
PHE: Public Heath England
pMCMC: particle Monte Carlo Markov Chain
