# Supplementary material for: Estimating the fitness cost and benefit of cefixime resistance in Neisseria gonorrhoeae to inform prescription policy: A modelling study
Source: PLoS Med. 2017 Oct 31;14(10):e1002416. doi: 10.1371/journal.pmed.1002416 (PMC5663337; doi:10.1371/journal.pmed.1002416)
Supplement: S1 Appendix — (PDF) [file pmed.1002416.s001.pdf]

**S1 Appendix. Model equations and stochastic simulations.** We developed a simple susceptible-infected-susceptible (SIS)-type stochastic compartmental model that describes the transmission and natural history of gonorrhea, as illustrated in Fig 1 with parameters summarised in Table 1. The population was assumed to be closed due to the short time period under consideration. A deterministic version of the model is described by the following differential equations, in order to illustrate the model dynamics.

$$\frac{dS(t)}{dt} = -\theta\frac{I(t)}{N}S(t) + \nu\big(A_{\text{sus}}(t) + \alpha A_{\text{res}}(t)\big) + \rho\big(T_{\text{sus;cef}}(t) + T_{\text{sus;oth}}(t) + (1-\phi)T_{\text{res;cef}}(t) + T_{\text{res;oth}}(t)\big) \tag{S1}$$

$$\frac{dU_s(t)}{dt} = \theta\frac{I_s(t)}{N}S(t) - \sigma U_s(t); s \in \{\text{sus}, \text{res}\} \tag{S2}$$

$$\frac{dA_{\text{sus}}(t)}{dt} = (1-\psi)\sigma U_{\text{sus}}(t) - \nu A_{\text{sus}}(t) \tag{S3}$$

$$\frac{dA_{\text{res}}(t)}{dt} = (1-\psi)\sigma U_{\text{res}}(t) - \alpha\nu A_{\text{res}}(t) + \phi\rho T_{\text{res;cef}}(t) \tag{S4}$$

$$\frac{dE_s(t)}{dt} = \psi\sigma U_s(t) - \mu E_s(t); s \in \{\text{sus}, \text{res}\} \tag{S5}$$

$$\frac{dT_{s;\text{cef}}(t)}{dt} = \pi(t)\mu E_s(t) - \rho T_{s;\text{cef}}(t); s \in \{\text{sus}, \text{res}\} \tag{S6}$$

$$\frac{dT_{s;\text{oth}}(t)}{dt} = \big(1-\pi(t)\big)\mu E_s(t) - \rho T_{s;\text{oth}}(t); s \in \{\text{sus}, \text{res}\} \tag{S7}$$

We used a stochastic version of the model described in Eqs S1-S7, time-discretized using Euler's method with time steps of 1 day. The process was initialised on 31 December 2007, with an initial number of asymptomatic infections $A_s(0)$ for $s \in \{\text{sus}, \text{res}\}$, and the remainder of the population being susceptible. Simulation proceeds through repeated iterations of the steps below for each day. Since the model is fully stochastic, it is possible for the outbreak to become extinct even when its basic reproduction number is greater than 1. Transition variables $d_1, \ldots, d_{17}$ are drawn from the following distributions:

$$d_1, d_2 \sim \text{Multinom}\left(S(t), \theta\frac{I_{\text{sus}}(t)}{N}, \theta\frac{I_{\text{res}}(t)}{N}\right) \tag{S8}$$

$$d_3, d_4 \sim \text{Multinom}\big(U_{\text{sus}}(t), \psi\sigma, (1-\psi)\sigma\big) \tag{S9}$$

$$d_5, d_6 \sim \text{Multinom}\big(U_{\text{res}}(t), \psi\sigma, (1-\psi)\sigma\big) \tag{S10}$$

$$d_7, d_8 \sim \text{Multinom}\big(E_{\text{sus}}(t), \pi(t)\mu, (1-\pi(t))\mu\big) \tag{S11}$$

$$d_9, d_{10} \sim \text{Multinom}\big(E_{\text{res}}(t), \pi(t)\mu, (1-\pi(t))\mu\big) \tag{S12}$$

$$d_{11} \sim \text{Binom}\big(A_{\text{sus}}(t), \nu\big) \tag{S13}$$

$$d_{12} \sim \text{Binom}\big(A_{\text{res}}(t), \alpha\nu\big) \tag{S14}$$

$$d_{13} \sim \text{Binom}\big(T_{\text{sus;cef}}(t), \rho\big) \tag{S15}$$

$$d_{14} \sim \text{Binom}\big(T_{\text{sus;oth}}(t), \rho\big) \tag{S16}$$

$$d_{15}, d_{16} \sim \text{Multinom}\big(T_{\text{res;cef}}(t), \phi\rho, (1-\phi)\rho)\big) \tag{S17}$$

$$d_{17} \sim \text{Binom}\big(T_{\text{res;oth}}(t), \rho\big) \tag{S18}$$

The compartments of the model are then updated as follows:

$$S(t+1) \coloneqq S(t) - d_1 - d_2 + d_9 + d_{12} + d_{13} + d_{14} + d_{15} + d_{17} \tag{S19}$$

$$U_{\mathrm{sus}}(t+1) \coloneqq U_{\mathrm{sus}}(t) + d_1 - d_3 - d_4 \tag{S20}$$

$$U_{\mathrm{res}}(t+1) \coloneqq U_{\mathrm{res}}(t) + d_2 - d_5 - d_6 \tag{S21}$$

$$E_{\mathrm{sus}}(t+1) \coloneqq E_{\mathrm{sus}}(t) + d_3 - d_7 - d_8 \tag{S22}$$

$$E_{\mathrm{res}}(t+1) \coloneqq E_{\mathrm{res}}(t) + d_5 - d_{10} - d_{11} \tag{S23}$$

$$A_{\mathrm{sus}}(t+1) \coloneqq A_{\mathrm{sus}}(t) + d_4 - d_9 \tag{S24}$$

$$A_{\mathrm{res}}(t+1) \coloneqq A_{\mathrm{res}}(t) + d_6 - d_{12} + d_{16} \tag{S25}$$

$$T_{\mathrm{sus;cef}}(t+1) \coloneqq T_{\mathrm{sus;cef}}(t) + d_7 - d_{13} \tag{S26}$$

$$T_{\mathrm{sus;oth}}(t+1) \coloneqq T_{\mathrm{sus;oth}}(t) + d_8 - d_{14} \tag{S27}$$

$$T_{\mathrm{res;cef}}(t+1) \coloneqq T_{\mathrm{res;cef}}(t) + d_{10} - d_{15} - d_{16} \tag{S28}$$

$$T_{\mathrm{res;oth}}(t+1) \coloneqq T_{\mathrm{res;oth}}(t) + d_{11} - d_{17} \tag{S29}$$
