# Supplementary material for: Estimating the fitness cost and benefit of cefixime resistance in Neisseria gonorrhoeae to inform prescription policy: A modelling study
Source: PLoS Med. 2017 Oct 31;14(10):e1002416. doi: 10.1371/journal.pmed.1002416 (PMC5663337; doi:10.1371/journal.pmed.1002416)
Supplement: S2 Appendix — (PDF) [file pmed.1002416.s002.pdf]

S2 Appendix. Derivation of basic reproduction numbers. We derive the equations for the basic reproduction numbers of the cefixime-resistant and susceptible strains by next generation method of calculation [1]. In order to calculate R_0 we must first define the matrices F and V for our model, where F is the matrix of partial derivatives of the rate of appearance of new infections into each compartment; and V is the matrix of partial derivatives of the net rate of transfer out of each compartment for reasons other than new infections. Both Jacobian matrices F and V are evaluated at the disease-free equilibrium ϵ_0, which in our model is:

$$\epsilon_0 = (S = N, U_s = 0, A_s = 0, E_s = 0, T_{s;p} = 0 : s \in \{\text{sus}, \text{res}\}; p \in \{\text{cef}, \text{oth}\}) \quad \text{(S30)}$$

R_0 is then calculated by finding the dominating eigenvalue of the matrix FV^{-1}. Using Eqns. (S1 - S7) we obtain:

$$F = \begin{array}{c} \\ U'_{\text{sus}} \\ U'_{\text{res}} \\ A'_{\text{sus}} \\ A'_{\text{res}} \\ E'_{\text{sus}} \\ E'_{\text{res}} \\ T'_{\text{sus;cef}} \\ T'_{\text{sus;oth}} \\ T'_{\text{res;cef}} \\ T'_{\text{res;oth}} \end{array} \begin{array}{c} \begin{array}{cccccccccc} U_{\text{sus}} & U_{\text{res}} & A_{\text{sus}} & A_{\text{res}} & E_{\text{sus}} & E_{\text{res}} & T_{\text{sus;cef}} & T_{\text{sus;oth}} & T_{\text{res;cef}} & T_{\text{res;oth}} \end{array} \\ \begin{pmatrix} \theta & 0 & \theta & 0 & \theta & 0 & 0 & 0 & 0 & 0 \\ 0 & \theta & 0 & \theta & 0 & \theta & 0 & 0 & 0 & 0 \\ 0 & 0 & 0 & 0 & 0 & 0 & 0 & 0 & 0 & 0 \\ 0 & 0 & 0 & 0 & 0 & 0 & 0 & 0 & 0 & 0 \\ 0 & 0 & 0 & 0 & 0 & 0 & 0 & 0 & 0 & 0 \\ 0 & 0 & 0 & 0 & 0 & 0 & 0 & 0 & 0 & 0 \\ 0 & 0 & 0 & 0 & 0 & 0 & 0 & 0 & 0 & 0 \\ 0 & 0 & 0 & 0 & 0 & 0 & 0 & 0 & 0 & 0 \\ 0 & 0 & 0 & 0 & 0 & 0 & 0 & 0 & 0 & 0 \\ 0 & 0 & 0 & 0 & 0 & 0 & 0 & 0 & 0 & 0 \end{pmatrix} \end{array} \quad \text{(S31)}$$

$$V = \begin{pmatrix} \sigma & 0 & 0 & 0 & 0 & 0 & 0 & 0 & 0 & 0 \\ 0 & \sigma & 0 & 0 & 0 & 0 & 0 & 0 & 0 & 0 \\ (\psi-1)\sigma & 0 & \nu & 0 & 0 & 0 & 0 & 0 & 0 & 0 \\ 0 & (\psi-1)\sigma & 0 & \alpha\nu & 0 & 0 & 0 & 0 & 0 & 0 \\ -\psi\sigma & 0 & 0 & 0 & \mu & 0 & 0 & 0 & 0 & 0 \\ 0 & -\psi\sigma & 0 & 0 & 0 & \mu & 0 & 0 & 0 & 0 \\ 0 & 0 & 0 & 0 & -\pi\mu & 0 & \rho & 0 & 0 & 0 \\ 0 & 0 & 0 & 0 & (\pi-1)\mu & 0 & 0 & \rho & 0 & 0 \\ 0 & 0 & 0 & 0 & 0 & -\pi\mu & 0 & 0 & \rho & 0 \\ 0 & 0 & 0 & 0 & 0 & (\pi-1)\mu & 0 & 0 & 0 & \rho \end{pmatrix} \quad \text{(S32)}$$

Therefore:

$$V^{-1} = \begin{pmatrix} \frac{1}{\sigma} & 0 & 0 & 0 & 0 & 0 & 0 & 0 & 0 & 0 \\ 0 & \frac{1}{\sigma} & 0 & 0 & 0 & 0 & 0 & 0 & 0 & 0 \\ \frac{1-\psi}{\nu} & 0 & \frac{1}{\nu} & 0 & 0 & 0 & 0 & 0 & 0 & 0 \\ 0 & \frac{1-\psi+\phi\pi\psi}{\alpha\nu} & 0 & \frac{1}{\alpha\nu} & 0 & \frac{\pi\phi}{\alpha\nu} & 0 & 0 & \frac{\phi}{\alpha\nu} & 0 \\ \frac{\psi}{\mu} & 0 & 0 & 0 & \frac{1}{\mu} & 0 & 0 & 0 & 0 & 0 \\ 0 & \frac{\psi}{\mu} & 0 & 0 & 0 & \frac{1}{\mu} & 0 & 0 & 0 & 0 \\ \frac{\pi\psi}{\rho} & 0 & 0 & 0 & \frac{\pi}{\rho} & 0 & \frac{1}{\rho} & 0 & 0 & 0 \\ \frac{(1-\pi)\psi}{\rho} & 0 & 0 & 0 & \frac{1-\pi}{\rho} & 0 & 0 & \frac{1}{\rho} & 0 & 0 \\ 0 & \frac{\pi\psi}{\rho} & 0 & 0 & 0 & \frac{\pi}{\rho} & 0 & 0 & \frac{1}{\rho} & 0 \\ 0 & \frac{(1-\pi)\psi}{\rho} & 0 & 0 & 0 & \frac{1-\pi}{\rho} & 0 & 0 & 0 & \frac{1}{\rho} \end{pmatrix} \quad \text{(S33)}$$

$$FV^{-1} = \begin{pmatrix} \theta\left(\frac{1}{\sigma} + \frac{1-\psi}{\nu} + \frac{\psi}{\mu}\right) & 0 & \frac{\theta}{\nu} & 0 & \frac{\theta}{\mu} & 0 & 0 & 0 & 0 & 0 \\ 0 & \theta\left(\frac{1}{\sigma} + \frac{1-\psi+\phi\pi\psi}{\alpha\nu} + \frac{\psi}{\mu}\right) & 0 & \frac{\theta}{\alpha\nu} & 0 & \frac{\theta}{\mu} & 0 & 0 & \frac{\theta\phi}{\alpha\nu} & 0 \\ 0 & 0 & 0 & 0 & 0 & 0 & 0 & 0 & 0 & 0 \\ 0 & 0 & 0 & 0 & 0 & 0 & 0 & 0 & 0 & 0 \\ 0 & 0 & 0 & 0 & 0 & 0 & 0 & 0 & 0 & 0 \\ 0 & 0 & 0 & 0 & 0 & 0 & 0 & 0 & 0 & 0 \\ 0 & 0 & 0 & 0 & 0 & 0 & 0 & 0 & 0 & 0 \\ 0 & 0 & 0 & 0 & 0 & 0 & 0 & 0 & 0 & 0 \\ 0 & 0 & 0 & 0 & 0 & 0 & 0 & 0 & 0 & 0 \\ 0 & 0 & 0 & 0 & 0 & 0 & 0 & 0 & 0 & 0 \end{pmatrix} \tag{S34}$$

Since the matrix is triangular, its eigenvalues are simply the diagonal entries, thus we obtain Eqns (1) and (2)

References

1. Diekmann O, Heesterbeek JAP, Metz JAJ. On the definition and the computation of the basic reproduction ratio R0 in models for infectious diseases in heterogeneous populations. Journal of Mathematical Biology. 1990;28(4):365–382. doi:10.1007/BF00178324.
